# Catalytic Approaches to Multicomponent Reactions: A Critical Review and Perspectives on the Roles of Catalysis

**DOI:** 10.3390/molecules27010132

**Published:** 2021-12-27

**Authors:** Brenno A. D. Neto, Rafael O. Rocha, Marcelo O. Rodrigues

**Affiliations:** 1 Laboratory of Medicinal and Technological Chemistry, University of Brasília, Chemistry Institute (IQ-UnB), Campus Universitário Darcy Ribeiro, Brasilia 70910-900, Brazil; rafaelrocha@unb.br (R.O.R.); Marcelo.Rodrigues@nottingham.ac.uk (M.O.R.); 2 School of Physics and Astronomy, Nottingham University, Nottingham NG72RD, UK

**Keywords:** catalysis, multicomponent reactions, mechanisms, solvent effects, stereocontrol

## Abstract

In this review, we comprehensively describe catalyzed multicomponent reactions (MCRs) and the multiple roles of catalysis combined with key parameters to perform these transformations. Besides improving yields and shortening reaction times, catalysis is vital to achieving greener protocols and to furthering the MCR field of research. Considering that MCRs typically have two or more possible reaction pathways to explain the transformation, catalysis is essential for selecting a reaction route and avoiding byproduct formation. Key parameters, such as temperature, catalyst amounts and reagent quantities, were analyzed. Solvent effects, which are likely the most neglected topic in MCRs, as well as their combined roles with catalysis, are critically discussed. Stereocontrolled MCRs, rarely observed without the presence of a catalytic system, are also presented and discussed in this review. Perspectives on the use of catalytic systems for improved and greener MCRs are finally presented.

## 1. Introduction

Catalysis plays a central role in chemistry and therefore is of vital importance to humankind [1]. Catalysis has existed since the dawn of civilization [2], and its paramount importance is beyond any doubt. The history of modern catalysis, involving attempts toward both a systematic investigation and understanding of this phenomenon, dates back to Jöns Jacob Berzelius in 1835 [3]. The IUPAC recommends the following definition: “*A catalyst is a substance that increases the rate of a reaction without modifying the overall standard Gibbs energy change in the reaction; the process is called catalysis. The catalyst is both a reactant and product of the reaction*” [4]. Catalytic approaches have been applied in several areas of chemistry, such as biofuel production [5], small organic molecules of interest [6], organic synthesis [7], sensor production [8], depollution [9], pharmaceutics [10] and many more [11,12,13].

**Scheme 1:**
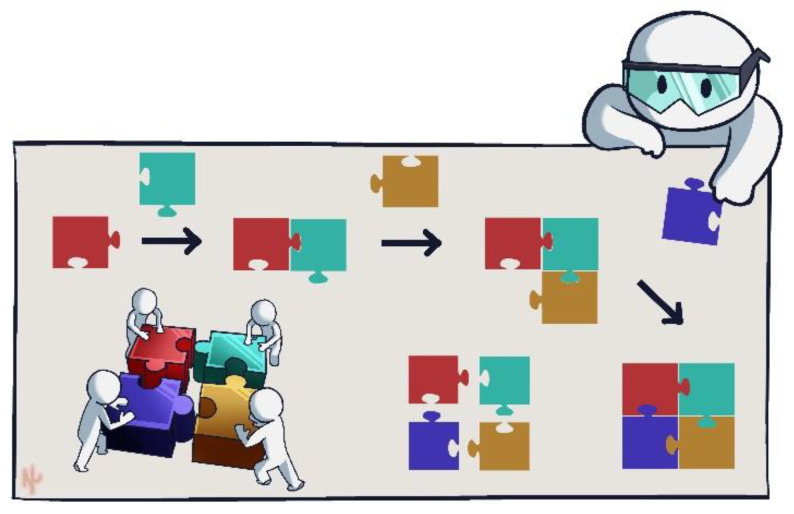
Multicomponent reactions in a pictorial view and the advantages over a linear synthesis.

Multicomponent reactions (MCRs) are currently in a prominent position in the organic toolbox (Scheme 1). The idea of bringing together three or more reagents in a one-pot transformation, preferentially added at once, incorporating most of the atoms from the reagents in the final structure, decreases the time and labor required in linear syntheses, which renders MCRs one of the most attractive possibilities regarding complexity and diversity, in addition to its prospects as a green approach. The importance of MCRs can be clearly noted by the large number of publications related to this field of research. Specialized and recently published reviews describing several features of MCRs are also available [14,15,16,17,18,19,20,21,22,23]. The oldest MCR described is credited to Adolph Strecker [24], who, in 1850, described his eponymous reaction, i.e., the Strecker amino acid synthesis or simply Strecker synthesis. The Strecker MCR is a reaction between amines, aldehydes and cyanide, affording α-aminonitriles (or α-aminocyanides) in a single one-pot operation, which, in turn, can be hydrolyzed to produce amino acids [25].

The modern history of catalysis (1835) and the history of MCRs (1850), as outlined, are therefore intimately connected. Several catalytic methodologies based on very different strategies, using homogeneous or heterogeneous conditions, metal- or organocatalysts, combinations of these methods/conditions and a plethora of different approaches, have been described [26,27,28] for most if not all known MCRs, although several multicomponent transformations may proceed as uncatalyzed reactions, as is discussed below. Despite the huge importance of catalysis in MCRs, the roles of catalysis in the promotion of such reactions are barely discussed. At first glance, one may consider catalysis as a tool to achieve yield improvements and shorter times, but as we intend to show herein, the multiple roles of a catalyst in MCRs go far beyond these two attributes.

The development of new catalytic systems, which are sometimes more seemingly random catalyst screening, may benefit from an in-depth investigation of the multiple roles of catalysis in multicomponent transformations. Reaction pathway selections, product formation without byproducts, lower energies to perform the reaction, waste generation prevention, use of equimolar reagent quantities in the reaction to maximize green indicators and stereocontrol are just a few features that are likely to ensue under appropriate catalyzed conditions of multicomponent synthesis.

In this tutorial review, we discuss and exemplify the roles of catalysis in MCRs. Thousands of articles are currently available and describe a plethora of catalytic systems applied to MCRs, but they lack critical investigation on how the presence of a catalyst influences selectivities, reaction pathways (mechanisms) and many other issues. These articles are outside the scope of this review and are cited only as exceptions to clarify, enrich or expand the discussion.

## 2. Mechanistic Considerations of MCRs and Catalysis Roles

**Scheme 2:**
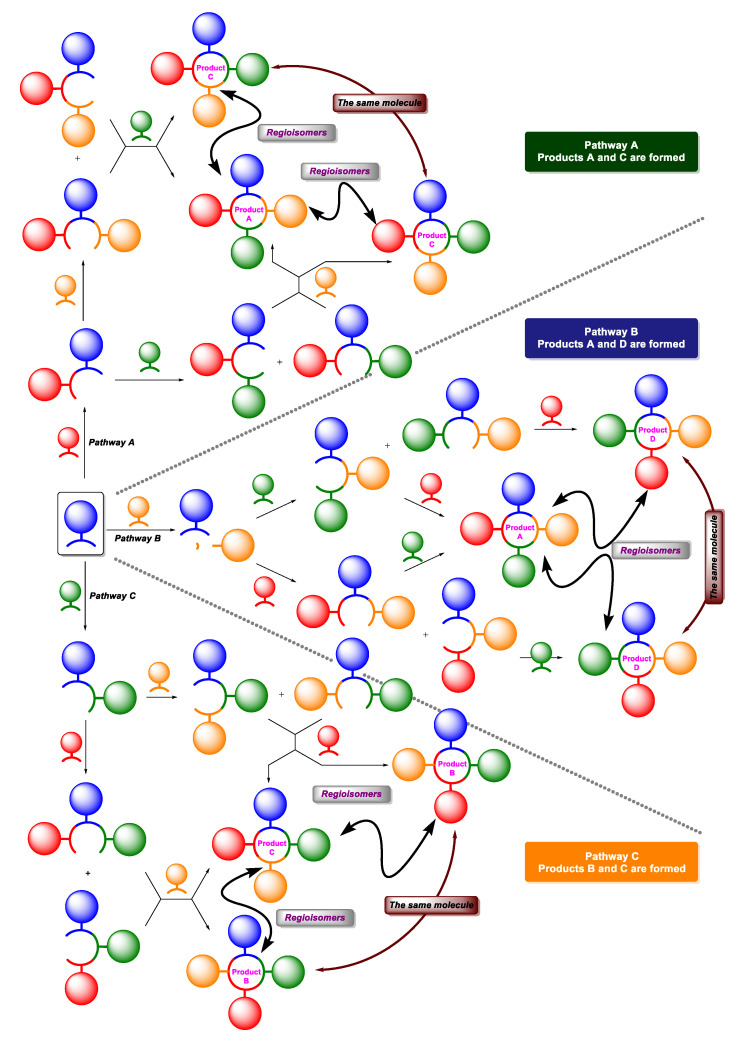
Pictorial view of a four-component MCR and the possibility of concurrent (and concomitant) reaction pathways. Note the possibility of diastereoisomer (regioisomer) formation. Enantiomer formation has been omitted for clarity. Side reactions are also a possibility.

MCR mechanism studies pose a huge challenge, as we have highlighted elsewhere [29]. MCRs typically have two or more mechanistic pathways (see a pictorial view in Scheme 2); hence, the possibility of concurrent but usually concomitant reaction pathways makes the investigation of such transformations a tricky and laborious task. Routine methods applied for investigating MCR mechanisms usually do not return detailed and expected information, so the combination of several methods is commonly required to obtain useful evidence to support a rationale of the transformation, as pointed out by Ivar Ugi [30].

At this point, as depicted in Scheme 2, it is possible to envisage one of the most important roles of catalysis in MCRs, that is, to select one reaction pathway among all theoretical possibilities to perform the transformation. In the current example (Scheme 2), a hypothetical four-component MCR can initiate through three different reaction pathway possibilities that may be operating at the same time and affording distinct reaction species or the same intermediates. Some reaction intermediates of the same structure can be formed at different stages of the transformation, depending on the reaction pathway. Besides the probability of side reactions (not shown in Scheme 2), one should also consider the possibility of forming diastereoisomers (e.g., regioisomers, as seen in Scheme 2) and enantiomers (not shown). The presence of an appropriate catalyst, however, may aid in the reaction pathway selection, thus allowing the planning and prediction of better reaction conditions to perform the MCR, as well as stereocontrol. By using a suitable catalytic system to conduct the MCR, it is therefore theoretically possible not only to select the reaction pathway but also to control diastereo- and enantioselectivities of the transformation.

**Scheme 3:**
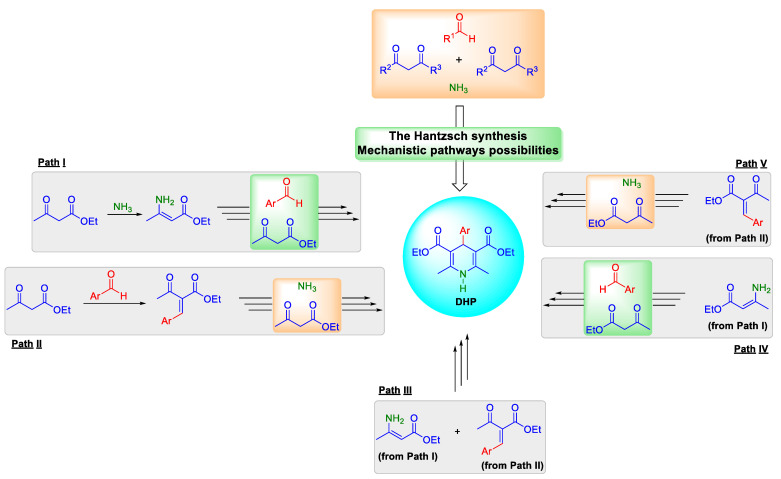
Hantzsch ester (DHP) formation and the five currently debated mechanistic pathway possibilities. Note that the mechanisms are shown in their simplified versions.

One example of a widely used MCR is the Hantzsch ester synthesis. There are five accepted possibilities to undertake the Hantzsch ester formation (1,4-dihydropyridine derivatives—DHPs), as we reviewed elsewhere [31]. As depicted in Scheme 3, the reaction pathways may take place in a concurrent manner. An in-depth mechanistic evaluation of the Hantzsch MCR and its competing mechanisms was achieved by using ESI-MS(/MS) to depict the preferential catalyzed (Bronsted acid-catalyzed version) reaction pathway [32]. Side reactions are also expected in the Hantzsch MCR [31]. In the presence of a catalyst, however, it is possible to considerably improve the reaction efficiency (higher yields), as we [33] and many groups [34,35,36,37,38,39,40] have already demonstrated, as well as select a preferred reaction pathway and avoid this large number of competing mechanisms. Under optimized catalytic conditions, side reactions can also be avoided or favored [41,42], depending on the interest, as recently demonstrated for the Hantzsch MCR [43] and for a Hantzsch-like [44] reaction.

**Scheme 4:**
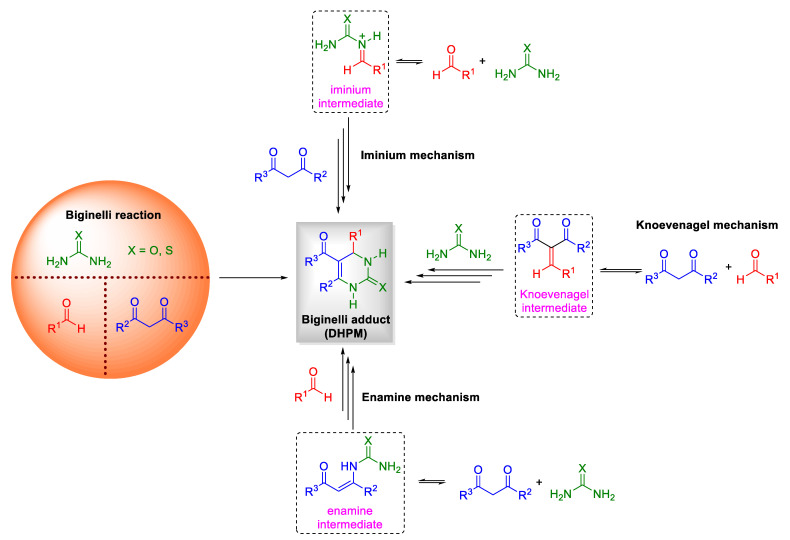
Currently accepted simplified mechanisms of Biginelli MCR, i.e., the iminium, the Knoevenagel and the enamine mechanisms. Note that their names are given after the first intermediate formed.

The Biginelli MCR, described in 1891 by Pietro Biginelli [45], is the first catalyzed multicomponent transformation reported. Initially, the reaction and an early “mechanistic investigation” were carried out as catalyst-free versions, but Biginelli himself discovered the beneficial effect on yields that a catalyst could exert over his eponymous reaction. When Bronsted acids (e.g., HCl) were used to facilitate DHPM formation (dihydropyrimidin-2(1*H*)-ones or -thiones, also known as Biginelli adducts), better yields were obtained in shorter times, as nicely reviewed elsewhere [45]. This important MCR has three currently accepted (and hotly debated) reaction pathways to obtain DHPM derivatives (Scheme 4). The intermediates observed in the Biginelli reaction are found in complex equilibria, as we [46,47,48,49,50] and others [51,52,53,54,55] have demonstrated. Preformation of one of these three initial intermediates is no guarantee that the mechanism will follow the expected reaction route due to the equilibria noted in their formations. The catalyst-free mechanism is known to proceed through the three possibilities shown in Scheme 4, as we demonstrated [48]. In another breakthrough work [56], the preference was demonstrated for the iminium mechanism in the presence of Bronsted or Lewis acid catalysts. The reaction efficiency was improved under catalyzed conditions and by selecting the appropriate solvent [56]. These examples showcase the catalyst’s role in selecting a preferred reaction pathway and also point to the implications of this fact for the enantioselectivities associated with this reaction, as is discussed in due course.

## 3. Catalyst-Free MCRs and Reasons to Use Catalysts

As precisely described by Orru and co-workers [57], “*Catalysis is at the heart of the green chemistry philosophy*”. Catalysis has indeed proven to be the most important tool to further MCRs, and its use is the least advantageous for most multicomponent transformations. It is general knowledge that there are dozens of reactions that can be performed with high yields under catalyst-free conditions. When the MCR arena is considered, however, these reactions are clearly rare exceptions [58,59,60,61]. Isocyanide-based MCRs are sometimes performed in the absence of catalysts [62], but their catalytic versions proved to have many advantages. Some catalyst-free MCRs have been induced by alternative conditions, such as light-promoted reactions [63], microwave irradiation [64] and a few others [65,66,67,68], but catalysis effects rather than these conditions are of interest in the current article. Some MCRs have been labeled “catalyst-free”, but they have also been elegantly questioned [69], whereas other so-called “catalyst-free” MCRs could not be reproduced at all. Low yields were indeed reported for their non-catalyzed versions under several distinct conditions [70,71,72,73,74,75]. Besides this reproducibility issue, which can sometimes be a major problem for MCRs, severe limitations can also be observed in the absence of a catalytic system.

As pointed out, the role of catalysis goes far beyond higher yields and shorter reaction times. Attempts to control the reaction pathway are not the only benefit of using catalysis. Another important feature of MCRs is the inherent green aspect of this class of reactions, as reviewed elsewhere [57]. In many cases, however, the reaction requires the use of a large excess of one of the reagents and, sometimes, at least two reagents are used in excess [76,77,78,79]. Under catalyzed conditions, this issue may be circumvented, or the problem may be considerably reduced. Waste generation prevention is known to be one of the main considerations related to green chemistry. Avoiding increases in waste levels is among the major advantages in the use of MCRs, and, in this sense, multicomponent transformations requiring excess reagents are contrary to this principle, although it may be necessary for some reactions.

**Scheme 5:**
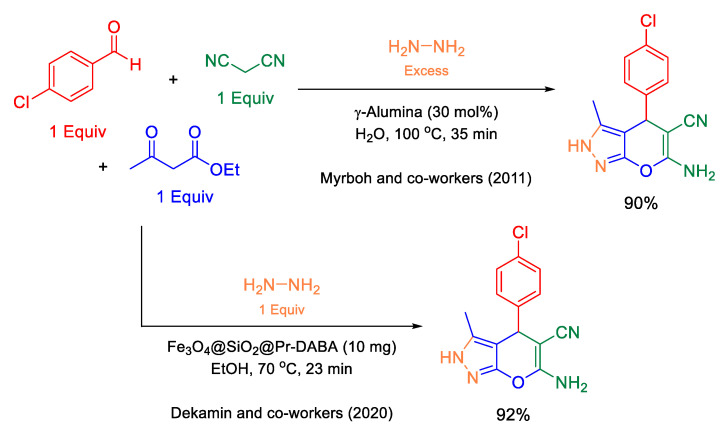
Catalytic four-component reaction exemplifying some catalysts’ roles.

Some MCRs have been described, for instance, using equimolar amounts of reagents and with high yields, with the application of a catalytic system developed for this purpose, especially for three- [80,81,82] and four-component [83,84,85] reactions. Reagent excess is clearly contrary to the green features expected for MCRs, and this issue should not be ignored, as noted in many available works. One example exemplifying this issue can be seen in Scheme 5.

Scheme 5 describes a four-component transformation applied in the synthesis of pyrano[2,3-c]pyrazole derivatives. Low yields (or traces) of the final adduct were observed under several catalyst-free conditions (see cited references). Efforts to develop catalyzed conditions have, however, been dedicated [86,87,88,89] to this important MCR transformation. Many of these catalytic systems required at least one reagent in excess [90,91,92,93,94], thus working against the desirable green and sustainable conditions anticipated for MCRs. As shown in Scheme 5, the application of catalytic conditions at 100 °C using one of the reagents in excess (hydrazine in this case) afforded the pyranopyrazole heterocyclic at 90% after 35 min of reaction [95]. Some features of the transformation, such as high catalyst amounts, reagent excess, elevated temperature and longer reaction times, were observed, even when a catalyst was used to perform the reaction. This fact should be enough to convince the reader of the challenges in executing MCRs in ideal conditions and the importance of developing more efficient catalysts. In this context, in another work [96], the development of a new and more efficient catalytic system allowed the reaction to be carried out with a relatively low catalyst amount, lower temperature, shorter times and equimolar use of the reagents; i.e., no reagent excess was required.

The effect of temperature was determined to be critical in many reactions. MCRs also experience considerable temperature effects. Energy efficiency is directly associated with the reaction temperature (and/or pressure). Reactions conducted at elevated temperatures or pressures are contrary to the green chemistry principle. For some MCRs, heating is imperative, whereas for others, it is just a matter of productivity associated with the energy demand. MCRs carried out under high-pressure conditions are not commonly described, although some examples can be found in the literature [97,98,99,100,101]. Considering that MCRs typically have sequences of elementary equilibrium stages and that thermodynamic adduct formation, or release of stable subproducts (e.g., water), favors the shifting of these stages toward the final desired adduct, high-energy-demanding multicomponent transformations can be avoided by employing catalysis. Side reactions can be circumvented and better diastereo- or enantiocontrols can be favored with the application of more amenable energy conditions during the transformation.

**Scheme 6:**
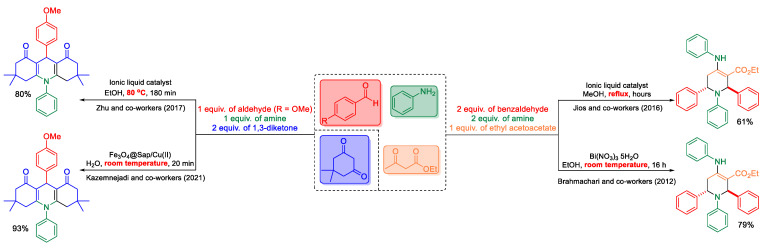
Catalysis effect on the reaction temperatures indicating the importance of developing catalytic systems toward more amenable reaction conditions. (Left) Catalyzed MCR for the synthesis of an acridine-1,8-dione derivative. (Right) Synthesis of densely functionalized piperidine derivative employing a catalyzed five-component MCR.

Several multicomponent transformations are performed at nearly 80 °C [102,103,104,105,106,107,108]. It is not rare for such reactions to be conducted at temperatures up to 100 °C [109,110,111,112,113] or even up to 120 °C [114,115,116,117,118,119]. Although these works are important contributions, it is highly desirable to use lower temperatures, especially bearing in mind enantio- and diastereocontrol, as is discussed in a separate section. MCRs carried out at room temperature are highly sought after, especially for saving energy and the prospect of future industrial applications for this class of reactions. Catalysis is therefore indispensable for MCRs performed at more amenable temperature conditions. Some elegant examples of MCRs have been performed at nearly room temperature (≈25 °C), and the presence of a catalyst proved to be essential to the success of the transformation at lower temperatures [120,121,122,123,124,125,126]. It is important to bear in mind that many reagents are less stable and less reactive than those typically used in model reactions characteristically described during the initial evaluation of an MCR. In this sense, a temperature decrease in the presence of a catalytic system has considerable advantages over non-catalyzed versions. Scheme 6 shows examples of catalysis efficiency in lowering the reaction temperatures of two different MCRs [127,128,129,130].

The synthesis of an acridinedione derivative was conducted, for instance, at 80 °C for a period of 180 min using ionic liquids as catalysts for the MCR transformation [127]. The adduct was obtained in good yield (80%), despite the challenges in completing this MCR. Several works [131,132,133] shed light on the synthesis of acridinedione scaffolds in catalyzed MCRs. The reaction in the absence of a catalytic system, even at higher temperatures, afforded the final adduct in very low yields (traces) [127]. An efficient catalytic system was, however, developed to perform the reaction at room temperature [128], and thus, the MCR adduct was obtained in high yields (93%) and in a short reaction time (20 min), as described in Scheme 6. In the second reaction, which is a different example of a five-component MCR transformation, densely functionalized piperidine derivatives were achieved (Scheme 6). Under reflux and in the presence of ionic liquid catalysts [129], the piperidine derivative was obtained in 61% yield (Scheme 6). The catalyzed version of this reaction has several advantages, as noted in a few reports [134,135,136]. The development of more efficient catalytic systems allowed the functionalized MCR adduct (Scheme 6) to be obtained at room temperature, i.e., a significant temperature decrease, when a bismuth catalyst was employed to promote the reaction [130].

## 4. The Combined Role of Catalysis and Solvent Effects in MCRs

Solvent effects have proved to be vital for the success of a plethora of reactions. Most industrial processes require the use of an appropriate solvent to succeed, as reviewed elsewhere [137]. A recent review also highlights the importance of using green and sustainable solvents in chemical processes [138]. Considering that there is an alignment between MCRs and green chemistry principles, the use of toxic and hazardous solvents for multicomponent transformations may be considered a “green herring”. Therefore, this means that when a report complies with one or two (or more) principles of green chemistry, it should not necessarily be labeled “a green and/or sustainable transformation” if some issues of the work do not diminish the current constraints that hinder MCR progress toward becoming a greener tool. The use of such solvents and conditions, even under catalyzed and amenable conditions, may in fact provide misleading conclusions. It is understandable that, sometimes, these solvents are indispensable, and it is not our goal to criticize any of these reports, but one should bear in mind that clean chemical technologies are the ultimate goal. Some [139] have already cross-examined issues related to green chemistry reports and pointed out their strengths and weaknesses in the context of limited advances when the concept is not properly applied.

Many MCRs have already been described [140,141,142,143,144] under solvent-free conditions, and this topic was reviewed elsewhere [145]. Although solvent-free MCRs may be interesting, multicomponent synthesis typically demands a solvent to proceed. Solvent screening in MCR reports is often described, but with no actual investigation of the solvent effect. Solvent effect investigations, rather than just solvent screening, are perhaps the most neglected topic in the MCR field. A few works, however, have demonstrated the beneficial role of the solvent’s effect in the reaction and discussed its combined role with catalysis, as discussed in this section.

**Scheme 7:**
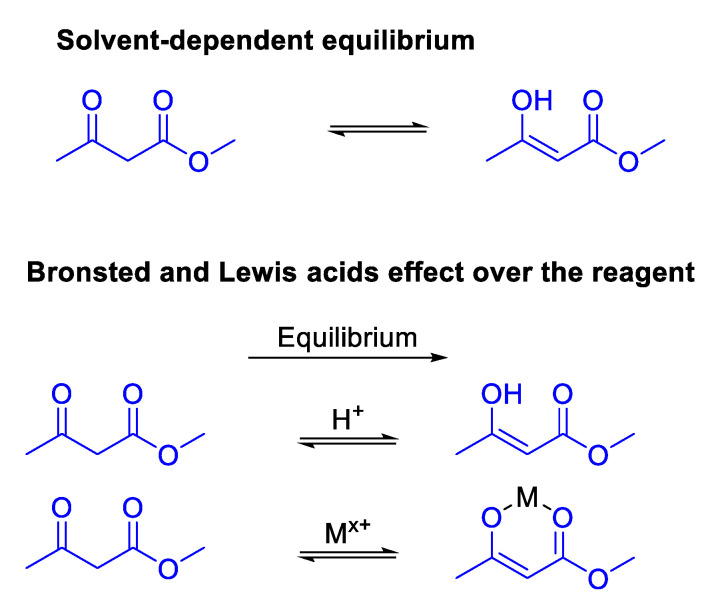
Solvent effect and catalysis associated with the good productivity of the Biginelli reaction as a consequence of enol tautomer formation/restoration of the 1,3-dicarbonyl derivative.

Sherwood and co-workers [56] did a good job of elucidating the solvent effect associated with the catalytic effect on the Biginelli reaction (also see Scheme 4). The solvent had a vital role in restoring the enol tautomer of the 1,3-dicarbonyl reagent to further the reaction productivity. As gauged by the Kamlet–Taft descriptors [146,147,148,149,150], the analysis of π* (dipolarity and polarizability) revealed a positive relationship between the reaction productivity and the solvent choice (Scheme 7) for the catalyzed reactions. The results indicated that the enol form was the reactive species, instead of the keto tautomer. In spite of the catalytic system (Lewis- or Bronsted-catalyzed reactions), the solvent proved to be fundamental in restoring the reactive tautomer and in furthering the reaction, thus highlighting the combined role of the solvent and catalysis.

Following this work [56], our group [48] demonstrated the pure solvent effect on the Biginelli reaction and the valuable role of catalysis combined with a solvent. Enol restoration was experimentally demonstrated, whereas DFT calculations indicated that enol reactivity was far higher than that of its keto tautomer. By combining the proper choice of a solvent and a catalyst, actual improvements could be observed. In a recent work [151], the use of palm oil with a Bronsted acid catalyst returned good results. The work developed a sustainable catalytic system using a bio-based alternative solvent and proved to be in accordance with the other two works indicating that tautomerism to the enol form was required for the reaction and the importance of both solvent and catalysis. Kamlet–Taft descriptors for some oils were determined and indicated their efficiency in encouraging enol tautomer formation. An outstanding work [152] described an in silico approach to obtain calculated Kamlet–Abboud–Taft solvatochromic parameters, and the methodology was evaluated in multicomponent heterocycle synthesis, thus providing new opportunities for using green solvents associated with more efficient catalysts.

**Scheme 8:**
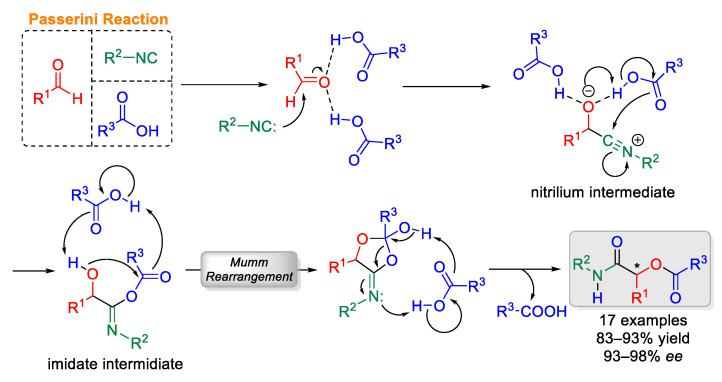
The proposed Passerini MCR mechanism.

The solvent effect on the Passerini MCR was investigated by DFT calculations (Scheme 8) and determined to be a key parameter for the reaction’s success [153]. In spite of the discussion surrounding the reaction mechanism associated with this multicomponent synthesis [154], Passerini adduct formation is dependent on the solvent, as we reviewed elsewhere [31]. In [153], nitrilium stability in solution was proposed, that is, an opposite view of the accepted thoughts regarding the Passerini reaction. The absence of H-bond networks when aprotic solvents were used encouraged Passerini adduct formation, whereas solvents such as methanol (polar and protic) proved to be responsible for a higher energy barrier during the rate-determining step of the transformation. Analyzing the proposed mechanism in [153] (Scheme 8), it is fair to suppose that H-bond formation takes place with carboxylic acid, especially in nonprotic solvents, since no competition for H-bond formation would be occurring. When additional quantities of this reagent were added to catalyze the multicomponent transformation, DFT calculations suggested a considerable lowering of the energy barrier in the appropriate solvent, thus pointing firmly to an associated effect of both the catalyst and solvent.

**Scheme 9:**
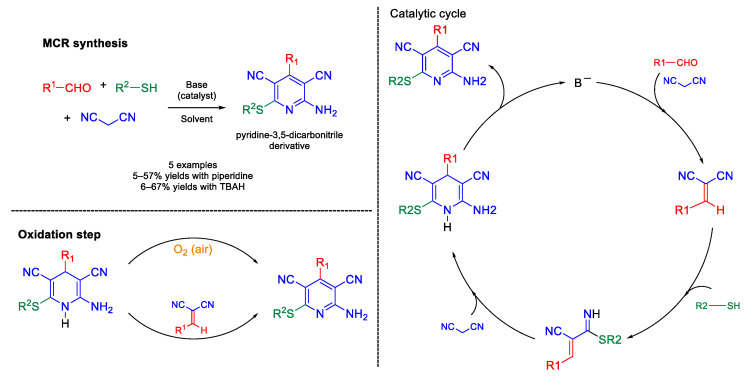
MCR synthesis and simplified mechanism for obtaining pyridine-3,5-dicarbonitrile derivatives. The reaction outcome was associated with the solvent and the base catalyst, indicating that both had a combined role in the reaction’s success.

The proper choice of a solvent and of a base as the catalyst proved to be essential for the outcome of an MCR applied to obtain pyridine-3,5-dicarbonitriles (Scheme 9) [155]. This outstanding investigation demonstrated that the reaction pathway and product formation were both basically dependent on the combination of the catalytic base and the solvent. Solubility differences in DMSO, ethanol and acetonitrile of the final adduct and 1,4-dihydropyridine (last intermediate in the catalytic cycle seen in Scheme 9) allowed the proposal of a solvent effect for the reaction. The intermediate was not soluble in MeCN, whereas the final adduct was completely soluble. Combining both the solvent and catalytic effects, significant differences were noted, especially in the oxidation step of the reaction (Scheme 9). The utilization of an ionic base promoted aerobic oxidation as the preferred reaction pathway. When an amine base was used, however, a H transfer mechanism from the 1,4-dihydropyridine derivative to the Knoevenagel intermediate was the dominant reaction pathway. The authors [155] also demonstrated the effect of reagent amounts on the reaction outcome.

**Scheme 10:**
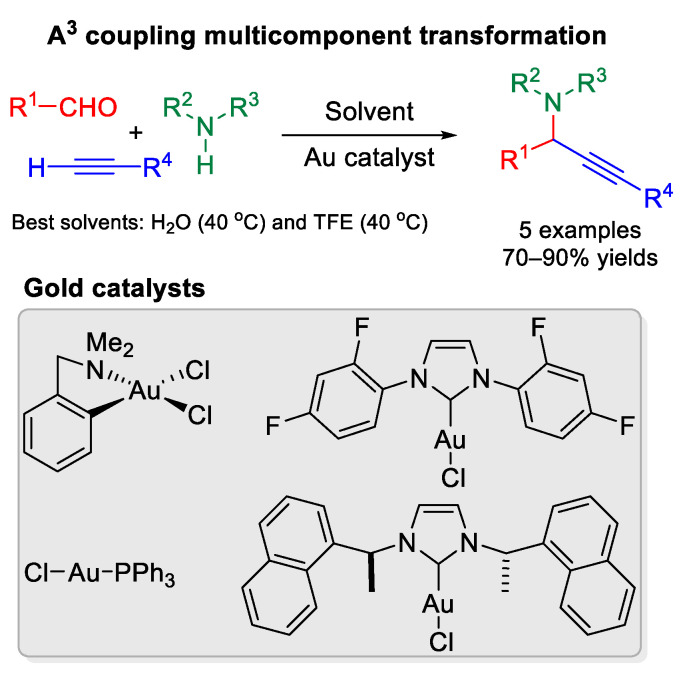
A^3^ coupling reactions were dependent on the solvent physicochemical properties and had a synergic effect with the gold catalysts.

An MCR known as A^3^ coupling (requiring an amine, an aldehyde and an alkyne) was efficient when conducted using mostly 2,2,2-trifluoroethanol (TFE) as the solvent, and it displayed a synergic effect in gold-catalyzed reactions (Scheme 10) [156]. This important MCR is largely used for preparing propargyl amines from aldehydes, alkynes and amines, as reviewed elsewhere [157,158,159]. Four gold complexes were tested as catalysts for this MCR, and different solvents required distinct temperatures to promote A^3^ adduct formation [156]. Reactions in the absence of a gold complex catalyst afforded no MCR adduct, whereas catalyzed transformations conducted in water (40 °C) proved to be as efficient as those performed in TFE (60 °C), requiring a lower temperature to produce the final MCR adduct. The beneficial solvent effects associated with the use of a gold catalyst were related to the physicochemical properties of the solvents, such as the known Reichardt solvatochromic parameters [160]. The authors highlighted the importance of the H-bonding network and the utilization of non-nucleophilic solvents [156]. These features are favorable toward iminium intermediate formation and its subsequent capture by the acetylenic gold σ-complex, i.e., a sequence of reactions favored due to the solvent effects and catalytic action of the gold complexes.

**Scheme 11:**
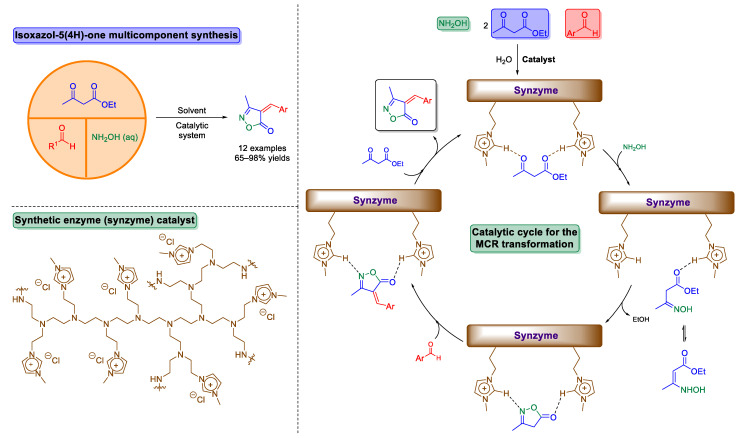
Multicomponent transformation for isoxazol-5(4*H*)-one (ISX) derivative synthesis catalyzed by a synzyme (synthetic enzyme) in water. The solvent and the catalyst had a synergic effect on productivity improvement. All intermediates were detected as charged species by high-resolution ESI-MS analyses, but the acidic H has been omitted for clarity.

We recently applied an MCR to the synthesis of isoxazol-5(4*H*)-one (ISX) derivatives, an important class of heterocyclics with potential and diverse biological activities [161,162,163,164,165,166], by using a synthetic enzyme (synzyme) that we had previously reported [167] as an efficient catalyst to perform ISX syntheses (Scheme 11) [168]. Some of these MCR adducts had fluorescent properties and could be applied in cell-imaging experiments as selective bioprobes. The mechanistic investigation of the reaction and the application of Kamlet–Taft descriptors to understand the transformation showed the combined role of solvent effects and catalysis. The best solvent to perform the reaction was water. Efficient transformation in aqueous media was possible considering the nature of the catalyst (with imidazolium cations and hydrophilic anions). An ionic liquid effect via activation of C=O was noted, and the keto tautomer seemed to be favored when trapped in the catalyst structure. In this sense, the electrophilic carbon was activated for the nucleophile addition and therefore furthered the reaction. ESI-MS(/MS) experiments revealed only one reaction pathway as the preferred one in the presence of the catalyst, although there were at least three possibilities. All key intermediates could be detected and characterized by collision-induced dissociation experiments.

The Kamlet–Taft analysis helped to explain the favorability of polar and protic solvents for performing the MCR and the preference for the depicted reaction pathway. This example of multicomponent synthesis showed the importance of studies on solvent effects and how these effects are associated with the catalytic action in the MCR. Although there are three possible mechanisms debated for ISX formation [169,170,171,172,173,174,175], the synthetic enzyme efficiently selected only one of them, as shown in Scheme 11. Water proved to be the best solvent, likely due to its hydrogen bond acidity (α) ability. The dependence of α indicated the beneficial effect on intermediate formation. Dipolarity and polarizability (π*) returned a relatively good correlation, indicating a valuable effect of the polar solvent over the polar (and charged) intermediates. A lack of any dependence on the β parameter (hydrogen bond basicity) could be observed for the MCR.

## 5. Catalysis in Stereoselective MCRs

Stereocontrol is a huge challenge for many types of reactions, and it is of special interest in the field of MCRs. Although many MCRs may afford one (or more) stereocenter, most of them are published as their racemic versions [176]. The possibility of diastereo- and/or enantiocontrol in one-pot multibond forming processes is an important feature of multicomponent transformations. As one may expect, catalysis has an unsurpassed role in this context, and most successful examples already reported for MCRs rely on the use of an appropriate catalyst [177,178,179,180,181,182,183,184,185,186]. Non-catalyzed multicomponent transformations have limited control in terms of selectivity, i.e., diastereo- and enantioselectivities. In the absence of a catalyst, any control will be dependent on the substrates’ nature and how they move closer to react, but with known limitations. Catalyzed MCRs, on the other hand, can be carried out with excellent control. Noncovalent interactions between catalysts and reagents/intermediates frequently play major roles in this context, as we recently highlighted [187].

**Scheme 12:**
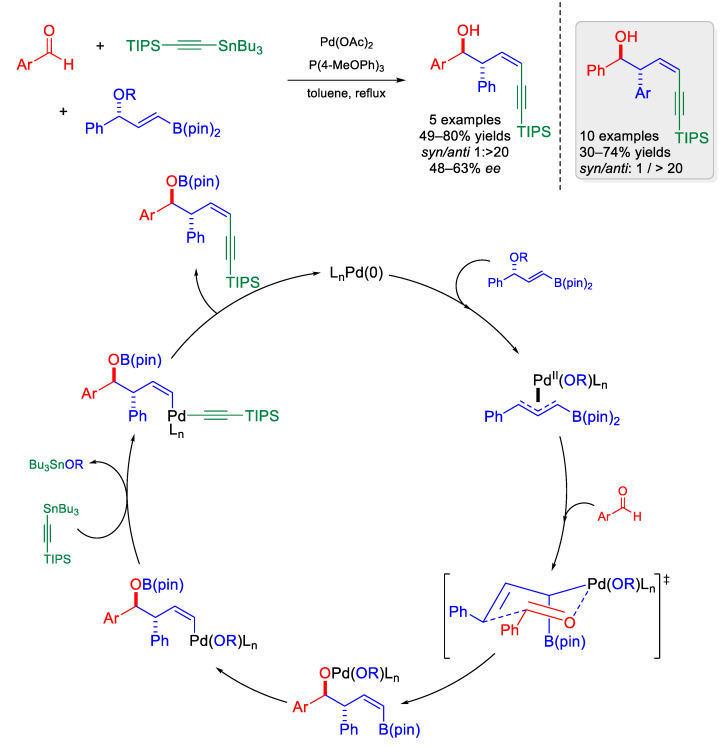
Pd-catalyzed multicomponent coupling reaction with high levels of diastereocontrol. The (*Z*) adduct was formed preferentially.

A recent example of complex multicomponent synthesis was described by Horino and co-workers [188]. In the reaction (Scheme 12), the authors observed high levels of (*Z*)-alkene stereocontrol. The catalyst proved to be essential for the MCR due to several features. Bifunctional conjunctive reagents afforded borylated π-allylpalladium species, which induced umpolung aldehyde allylation reactions in the presence of the catalyst. As expected, C-C bond formation and stereocontrol could be achieved exclusively in the presence of the palladium catalyst. The use of the palladium catalyst, as noted in Scheme 12, allowed several derivatives to be obtained. The metal catalyst exerted unique roles in two major steps of the transformation, thus indicating the efficiency of catalysis for the whole transformation, i.e., a feature that, in principle, is not possible for uncatalyzed reactions. The transition state proposed in the work indicates the origin of the stereocontrol and how the reagents are brought together by the metal catalyst during the preferential formation of the (*Z*) adduct (see Scheme 12).

**Scheme 13:**
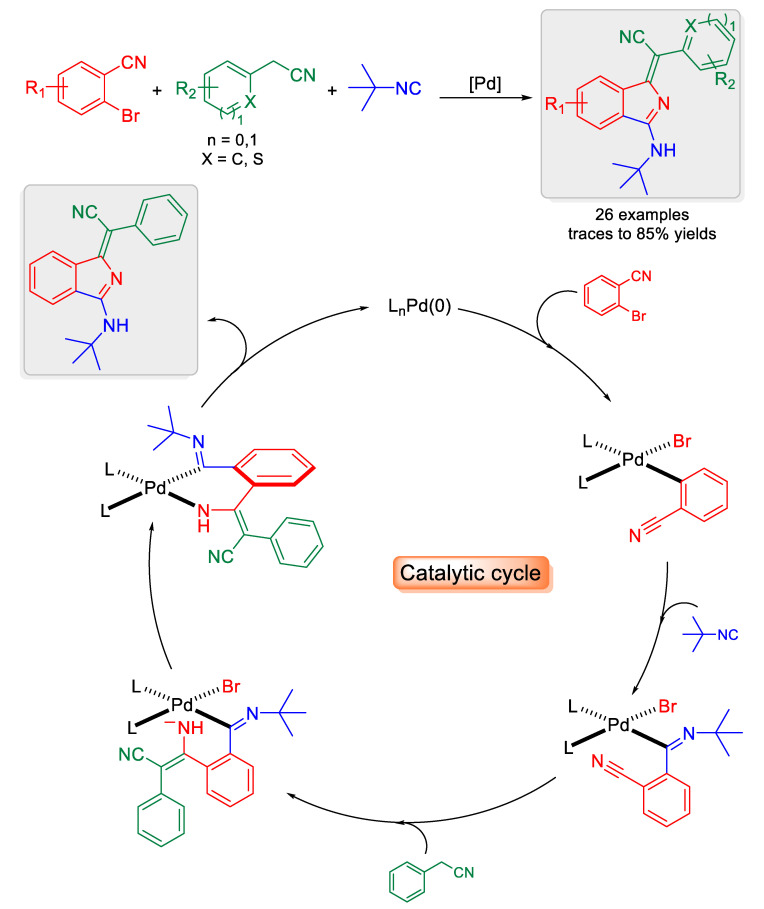
Triple C≡N activation in the catalyzed MCR applied in the synthesis of (*Z*)- or (*E*)-1*H*-isoindole with high stereocontrol.

In a different example, aggregation-induced emission (AIE) fluorophores could be synthesized using a Pd-catalyzed MCR by selective C≡N triple-bond activation. Triple activation of three different triple bonds could only be achieved by using an adequate catalyst and allowed the description of one unprecedented multicomponent synthesis. The reaction selectively afforded (*Z*)- or (*E*)-1*H*-isoindole derivatives (Scheme 13) [189]. In the presence of a catalytic system, the selectivity could be driven by different reactivities of electrophilic sites. The isocyanide could be activated via an insertion reaction, and, subsequently, a nucleophilic addition reaction to the nitrile group would give the next intermediate. Consecutive reductive elimination and isomerization reactions afford the desired AIE adducts. Some of the synthesized derivatives were applied as bioimaging probes and proved to be capable of staining lipid droplets inside live cells in a selective fashion. This landmark work [189] represents the power of catalyzed multicomponent synthesis and the vital catalyst effect with multiple roles to achieve the desired compounds with high stereocontrol. In this example, the efficiency of the metal catalyst is clear in some of the steps shown in the reaction mechanism (Scheme 13).

**Scheme 14:**
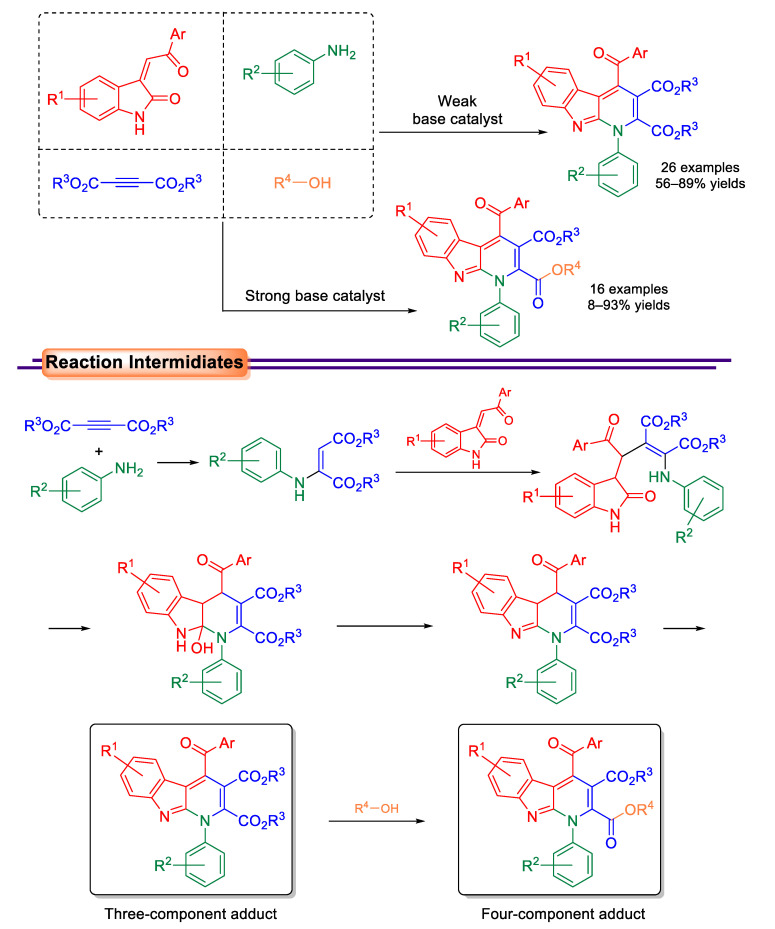
Three- or four-component pyrido[2,3-*b*]indole derivative synthesis. The catalyst’s strength determines whether it is a three- or four-component reaction. The last step of the four-component synthesis takes place in a high regioselective fashion.

A base-catalyzed multicomponent synthesis, with high regiocontrol, was performed using a three- or four-component version, depending on the selected catalyst [190]. The catalyzed reaction allowed for the access of polyfunctionalized pyrido[2,3-*b*]indoles (Scheme 14). In the absence of a base catalyst, only traces of the MCR adduct were obtained and only in the three-component version. The use of a strong base (*^t^*BuOK in this case) turned the multicomponent transformation into a four-component version. In the four-component reaction, a final MCR adduct with controlled regioselectivity in the final transesterification reaction, a feature not observed when weaker bases were tested, was obtained in high yields. Weak bases afforded exclusively final adducts, as expected for three-component transformations. A high level of regiocontrol was achieved only using the stronger base catalyst, and a series of compounds from the four-component reaction version was synthesized. This exemplifies the importance of tuning the catalyst’s properties and how catalysis is capable of affecting the outcome of an MCR.

**Scheme 15:**
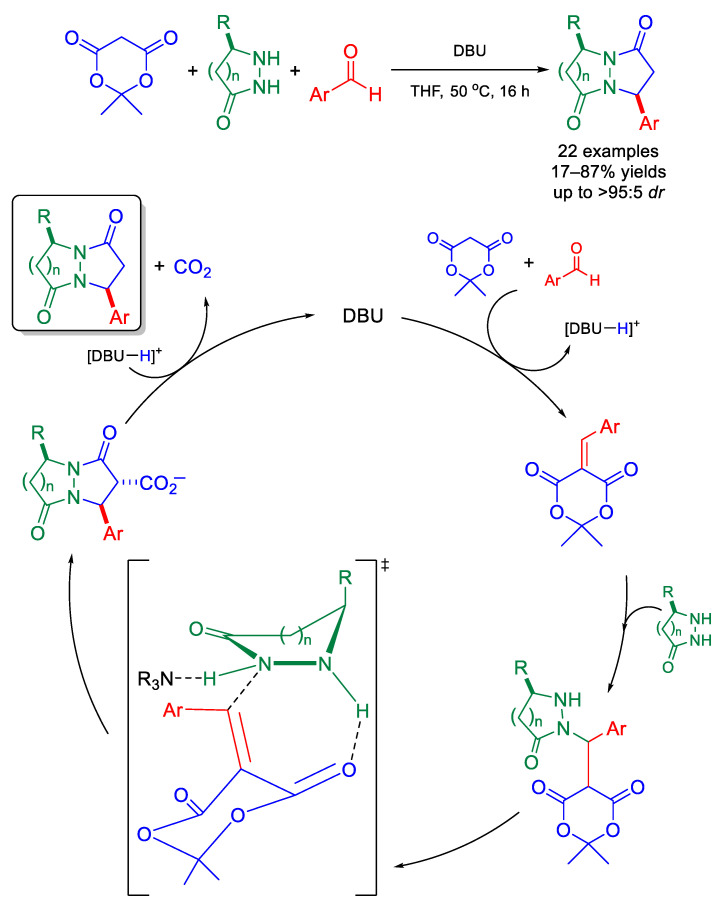
DBU-catalyzed stereocontrolled MCR for the synthesis of 1,6-diazabicyclo[4.3.0]nonane-2,7-dione derivatives.

A DBU-catalyzed MCR was successfully applied in the synthesis of 1,6-diazabicyclo[4.3.0]nonane-2,7-diones with high stereocontrol [191]. The desired adduct was obtained with a diastereoisomeric ratio up to 95:5 favoring the *cis* derivative in the model reaction. The regiocontrol of the reaction was an additional feature noted in the reaction mechanism (Scheme 15). The base catalyst also helped to favor a conformation in which the substituents are far from each other, thus facilitating the stereocontrol of the reaction. The mechanism was also investigated by ESI-MS(/MS) and pointed to the accuracy of the mechanistic proposition, as well as the catalyst’s roles in the MCR.

**Scheme 16:**
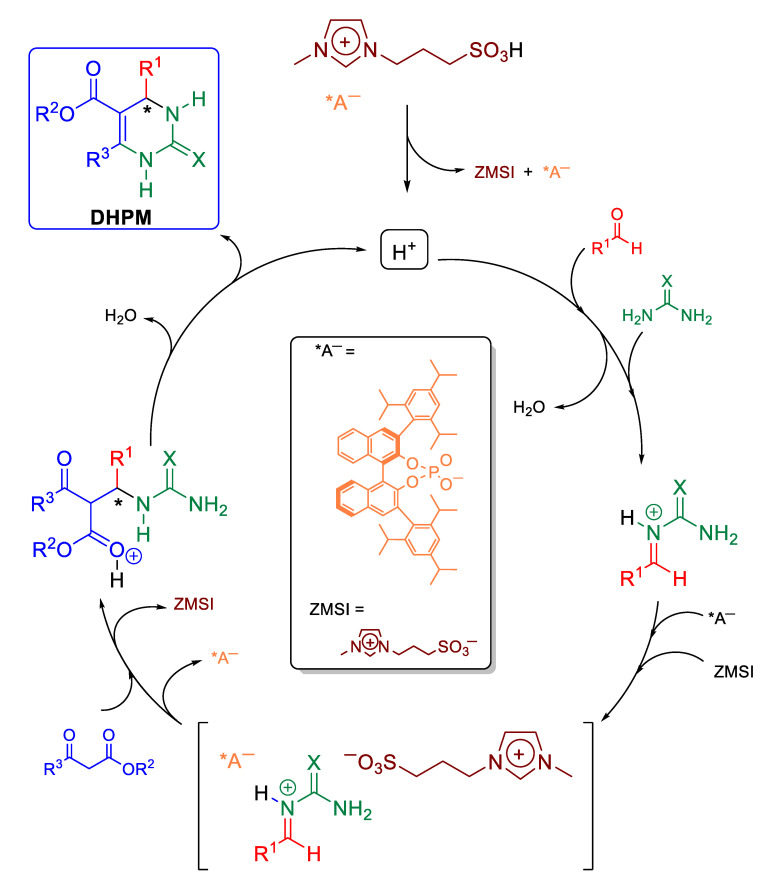
Chiral catalyst with triple role for enantiomeric control and reaction pathway selection.

An example of a multitask catalyst with enantiomeric control was recently reported by our group [192]. To perform the enantioselective synthesis of 3,4-dihydropyrimidin-2(1*H*)-one derivatives, it is necessary to use (i) a chiral inductor [193], (ii) a Bronsted (or Lewis) acid catalyst (or co-catalyst) [194] and (iii) an organic salt to improve enantiomeric excess [195]. A solvent may or may not be used for this specific multicomponent synthesis [145]. Included in the structure of the developed catalyst (Scheme 16) [192] was a chiral inductor, a Bronsted acid and, since it is a task-specific ionic liquid, an organic salt, thus making it a unique catalyst with a triple role in the transformation. As discussed in the article [192], the catalyst was efficient in promoting the synthesis and in exclusively selecting one reaction pathway (Scheme 16). High levels of enantiomeric control were noted due to a cage-like transition state for the favored enantiomer and with significant noncovalent interactions. The unfavored enantiomer had an open transition state with a higher energy barrier. The paramount importance and roles of catalysis in MCRs are clearly noted in this example.

## 6. Homogeneous and Heterogeneous Catalysis

Catalyzed MCRs may be carried out under homogeneous and heterogeneous conditions. Both conditions have advantages and limitations [26]. Our group demonstrated [50], for instance, some limitations on the recovery of the heterogeneous catalytic system for an MCR as a consequence of product precipitation. The final MCR adduct precipitated in the reaction medium and remained adsorbed in the heterogeneous catalysis, whereas in the homogeneous version, product separation was far less laborious. Some multicomponent syntheses, however, may be efficiently conducted under heterogeneous conditions with effective catalytic system recovery [196,197,198,199,200,201]. Metal–organic frameworks (MOFs) are, however, active, programmable and recyclable catalysts successfully applied in MCR syntheses, as recently reviewed elsewhere [202,203,204]. This comparison reinforces the advantages of each newly developed catalytic cycle and highlights the importance of new approaches.

When considering enantioselective MCRs, however, there is a huge gap between homogeneous and heterogeneous catalyzed reactions. A plethora of successfully performed multicomponent syntheses under homogeneous catalysis can be found in the literature [178,186,205,206,207,208,209,210,211,212,213,214,215,216,217,218,219,220,221], whereas heterogeneous reactions are not.

**Scheme 17:**
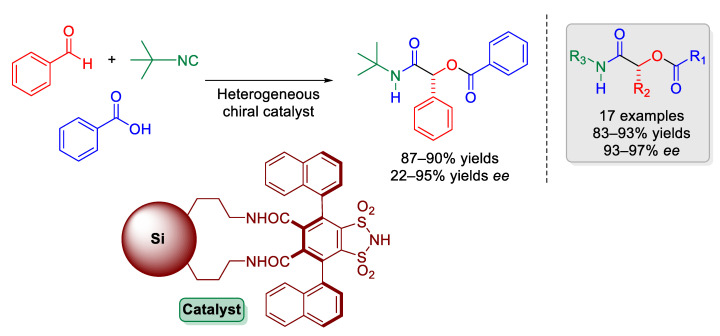
Enantioselective Passerini reaction using a chiral heterogeneous catalytic system.

One exception for enantioselective heterogeneous-catalyzed MCRs is the recently published work of Dughera and co-workers [222]. In their work, a chiral benzenedisulfonimide derivative was immobilized with 3-aminopropyl functionalized silica gel, affording the heterogeneous catalyst, which was successfully applied in an enantioselective version of the Passerini reaction (Scheme 17).

Several solvents and conditions were tested, but the reaction performed better using a deep eutectic mixture (urea/choline chloride) [222]. Numerous derivatives could be obtained with high yields (78–93%) and enantiomeric excesses (up to 92% for all substrates). The heterogeneous nature of the catalyst allowed its recovery and reuse in the multicomponent synthesis. Five cycles could be carried out, and both yields and enantiomeric excesses remained similar to those in the first cycle (i.e., nearly 95 and 94%, respectively). This work [222] certainly represents a huge advance in the use of heterogeneous catalysis to perform enantioselective MCRs. The limited number of available catalysts to carry out enantioselective multicomponent transformations under heterogeneous conditions is strongly indicative of challenges in the development of these catalytic systems. It also highlights a large window of opportunities for the development of new heterogeneous catalytic systems.

## 7. Concluding Remarks

The history of MCRs is intimately bound to catalysis. The huge development observed in the field of multicomponent syntheses is associated with the design and application of more efficient catalytic systems, which in turn are related to mechanistic knowledge of the multicomponent transformation. Catalyst-free MCRs are exceptions, and, in general, these and correlated expressions are no more than buzzwords. The advantages of catalyzed MCRs far exceed those of the non-catalyzed versions for almost all examples described.

The role of catalysis is not only to improve yields and shorten reaction times, but all selectivities, including a reaction pathway selection, are interconnected to catalysis. Several MCRs have at least two or more possible reaction pathways, which may be operating at the same time. Under catalyzed conditions, however, these reactions have a strong tendency to proceed through one preferential reaction pathway as a consequence of the catalytic action. This topic is of special interest when stereocontrolled MCRs are carried out.

Catalysis fosters the beneficial connection between green chemistry and multicomponent transformations, hence allowing, for instance, the use of equimolar quantities of the reagents in several examples and helping to reduce the generation of waste. In spite of the progress in reducing reagent excess for MCRs, there are still several reactions requiring at least one reagent to be used in excess to achieve better yields. This feature shows that there is still plenty of room for improving these reactions and shows the need for more rational designs of new catalytic systems to advance this field of research. A few works highlighted the importance of the combined role of catalysis and solvent effects for some MCRs. This is perhaps the most neglected topic of interest for multicomponent synthesis, i.e., in-depth evaluation of solvent effects over catalyzed multicomponent transformations.

Enantiocontrolled MCRs almost exclusively succeed when a catalyzed version of the reaction is performed. Diastereo- and enantiocontrols in the absence of a catalyst to guide the reaction are a few exceptions in the field of multicomponent synthesis, and most of the significant works achieved these goals under catalyzed conditions. Several examples are discussed in this review, exemplifying the vital roles of catalysis in conducting stereocontrolled MCRs. Homogeneous catalysts are far more used to perform enantioselective multicomponent transformations. This field of research still requires improvements, especially for heterogeneous catalysis.

In this tutorial review, we highlight both the essential and multiple roles of catalysis, aiming to improve MCRs. From the examples and discussions shown herein, we believe that there is no reasonable doubt regarding the vital importance of catalysis in MCRs and the challenges in the development of more efficient catalytic systems. We hope to have stimulated and challenged readers to explore new catalytic systems with improved performance in all fields of multicomponent transformations, especially because we believe that it is impossible to separate the future of these reactions and catalysis itself.

## Data Availability

Not applicable.
